# Improving Autonomous Vehicle Perception through Evaluating LiDAR Capabilities and Handheld Retroreflectivity Assessments

**DOI:** 10.3390/s24113304

**Published:** 2024-05-22

**Authors:** Ziyad N. Aldoski, Csaba Koren

**Affiliations:** 1 Department of Highway and Bridge, Technical College of Engineering, Duhok Polytechnic University, 61 Zakho Road, Duhok 1006, Kurdistan Region, Iraq; ziyad.nayef@dpu.edu.krd; 2 Department of Transport Infrastructure and Water Resources Engineering, Faculty of Architecture, Civil Engineering and Transportation Sciences, Széchenyi István University, Egyetem tér 1, 9026 Győr, Hungary

**Keywords:** autonomous vehicles, LiDAR, retroreflectometer, traffic sign visibility, road safety, road infrastructure

## Abstract

Road safety is a serious concern worldwide, and traffic signs play a critical role in confirming road safety, particularly in the context of AVs. Therefore, there is a need for ongoing advancements in traffic sign evaluation methodologies. This paper comprehensively analyzes the relationship between traffic sign retroreflectivity and LiDAR intensity to enhance visibility and communication on road networks. Using Python 3.10 programming and statistical techniques, we thoroughly analyzed handheld retroreflectivity coefficients alongside LiDAR intensity data from two LiDAR configurations: 2LRLiDAR and 1CLiDAR systems. The study focused specifically on RA1 and RA2 traffic sign classes, exploring correlations between retroreflectivity and intensity and identifying factors that may impact their performance. Our findings reveal variations in retroreflectivity compliance rates among different sign categories and color compositions, emphasizing the necessity for targeted interventions in sign design and production processes. Additionally, we observed distinct patterns in LiDAR intensity distributions, indicating the potential of LiDAR technology for assessing sign visibility. However, the limited correlations between retroreflectivity and LiDAR intensity underscore the need for further investigation and standardization efforts. This study provides valuable insights into optimizing traffic sign effectiveness, ultimately contributing to improved road safety conditions.

## 1. Introduction

Autonomous vehicles (AVs) represent a revolutionary advancement in transportation innovation, promising to significantly improve modern transportation systems’ safety, efficiency, and convenience. A fundamental prerequisite for the successful operation of AVs is the accurate perception and interpretation of environmental cues, emphasizing essential elements such as traffic signs. These signs serve as crucial visual indicators that convey regulatory, warning, and informational instructions to drivers and autonomous systems alike. Ensuring proper traffic sign visibility is essential to facilitate safe and efficient navigation for these vehicles [1].

Traffic sign visibility is a complex and multifaceted phenomenon influenced by various factors, including environmental conditions, lighting, sign placement, and the perceptual capabilities of the system interpreting the signs [2]. Understanding traffic sign visibility is paramount for effectively designing and operating AV systems. Traditional vision assessment methods predominantly rely on human perception and on-site observations, centering on enhancing the signs’ retroreflection coefficient (Ra).

However, in the context of AVs, there is a compelling need to integrate advanced technologies, notably LiDAR-based analysis, to provide real-time, data-driven insights into traffic sign visibility. Incorporating such technologies is imperative for overcoming traditional approaches’ limitations and ensuring AVs’ seamless integration into dynamic and complex traffic scenarios [3]. This paper advocates for a paradigm shift towards an interdisciplinary approach, combining conventional assessment methodologies with advanced technologies, to holistically address the intricate challenges associated with traffic sign visibility in autonomous driving technology.

This study integrates the previously developed comprehensive framework, which integrates portable retroreflectometer measurements and LiDAR-based analysis methodologies. Through this synergistic integration, the study sets out to achieve several objectives:

1. Evaluate the efficacy of traditional vision assessment methodologies:
  - Scrutinize the effectiveness of portable retroreflectometer measurements in assessing retroreflectivity, mainly focusing on the RA1 and RA2 sign classes.
  - Assess the contextual applicability of traditional vision assessment methodologies within the domain of AVs.
2. Investigate LiDAR-sensor-based assessments in the context of AVs:
  - Examine the capabilities and limitations of LiDAR-based analysis methodologies for assessing road sign visibility.
  - Integrate LiDAR and retroreflectometer methodologies to refine the evaluation approach.
  - Discern potential correlations and limitations between LiDAR intensity and handheld retroreflectivity coefficient measurements.
3. Provide valuable insights for road authorities:
  - Improve road traffic signs’ overall management and maintenance.
  - Tackle the critical safety issue of declining retroreflectivity values in road traffic signs.
  - Contribute to the enhancement of road safety standards.

These objectives collectively aim to address the critical safety issue of declining retroreflectivity values of road traffic signs. This is an important issue that needs to be addressed to improve road safety standards and effectively manage traffic signs, especially in the context of autonomous driving technology. The research integrates various methodologies by systematically analyzing LiDAR intensity data collected in diverse contexts and delineating the relationship between handheld retroreflectometer measurements. This approach allows for a comprehensive understanding of traffic sign visibility, considering different factors that may affect it. The overarching goal is to develop an evaluation approach that combines the strengths of each methodology, providing a nuanced and detailed understanding of traffic sign visibility in the specific context of autonomous driving technology. This integrated approach should yield valuable insights that will significantly contribute to the refinement and optimization of AV systems for safe and efficient navigation through diverse and dynamic traffic scenarios.

## 2. Related Work

Exploration of traffic sign visibility is critical for the safe operation of AVs and their surrounding environment. This paper delves into a comprehensive literature review, combining seminal studies, methodological frameworks, and insights to improve the accuracy of traffic sign detection in autonomous driving systems. The potential hazards of inadequate visibility of road equipment, particularly the crucial role of retroreflectivity, highlight the need for a thorough evaluation of visibility. This part evaluates traditional handheld retroreflectometer-based assessment approaches and their integration with LiDAR technology. Through carefully examining scholarly resources, the study systematically assesses the precision and efficacy of these methodologies, aiming to determine the most effective method for evaluating traffic sign visibility. Within this broader investigation, the paper underscores the increasing attention paid to optimizing AV navigation systems, specifically signage visibility, and the critical contribution of AV sensors in this context.

Academic discourse on retroreflection in traffic signs dates back to the 1930s when the American company Potters pioneered the use of glass beads to enhance brightness in cinema screens. This innovation was later applied to road markings and signs, although with limited efficiency. According to ref. [4], only 28% of the glass bead surface area is adequately reflective at favorable angles, resulting in light losses upon interaction with the glass surface. Paper [5] emphasizes the historical reliance on glass beads in early retroreflective sheeting and the introduction of a specialized glass surface to counter diminished reflectivity due to condensation. In a comprehensive study, ref. [6] utilized handheld retroreflectometers and vehicular camera systems to quantify retroreflection and traffic sign recognition. They found that the number of signs affixed to a post was a determining factor in recognition distance, shedding light on the factors that influence sign detection. Other studies have also explored the complex relationship between environmental variables and retroreflective attributes within traffic signs, including contamination, precipitation, temperature, and relative humidity; refs. [1,7,8] have contributed insights into the installation chronology, orientation, geographical disposition, color, and classification of traffic signs, all of which can impact road sign retroreflectivity.

Paper [9] studied predictive models for road traffic sign retroreflectivity, utilizing algorithms such as Random Forest and Artificial Neural Networks. Their research achieved high accuracy in classifying signs based on retroreflectivity levels, highlighting the importance of variables such as signage, sheeting class, and color. The results of their study offer valuable insights for improving the durability and functionality of road traffic signs. However, assessing retroreflectivity and chromatic characteristics in road traffic signs is a complex task, as there is no definitive threshold for determining operational obsolescence. This challenge has been observed in studies conducted in Sweden [10] and Hungary. In a separate study, ref. [11] examined the relationship between road attributes, traffic incidents, and traffic sign quality. Their findings revealed a direct correlation between traffic sign quality and the frequency of accidents. They suggested that improving traffic sign quality, especially on roads with high Average Annual Daily Traffic (AADT) and longer lengths, could reduce accidents.

Paper [12] provided analytical insights into predicting retroreflection degradation in traffic signs using the University of Zagreb’s Department for Traffic Signalization dataset. Their study emphasized the importance of linear models in forecasting retroreflection degradation, emphasizing their role in strategic maintenance efforts and improving road safety. The literature has extensively discussed the effectiveness of LiDAR in detecting roads, traffic signs, and markings in AV environments [13]. Intensity analysis, a crucial method for optimizing traffic sign visibility through LiDAR, is essential for determining visibility and contrasting various environmental conditions [14]. Moreover, paper [15] referenced the work of [16], which introduced a LiDAR point cloud reflectivity method for detecting and categorizing vertical traffic signs.

The reflectivity characteristics of surfaces significantly impact the accuracy of LiDAR devices, which can pose challenges for detection, particularly on surfaces with high reflectivity [17]. Additionally, the functionality of AV sensors can be affected by challenges in detecting objects with similar colors and light reflectivity, as pointed out by ref. [18]. To address these challenges, research has explored various methodologies for traffic sign recognition in AVs, including color-based categorization, shape-based detection, deep learning methods, and edge detection techniques [2]. Furthermore, investigations have been conducted to understand the impact of environmental factors, such as lighting, automotive paints, target angles, distances, and surface conditions, on the performance of LiDAR and cameras in AVs [19]. Reference [20] conducted empirical tests to examine the impact of diverse weather conditions on LiDAR detection performance in Korean road traffic signs. Moreover, ref. [21] underscores the significance of collaborative data collection from multiple sensors for 3D object detection in autonomous driving scenarios. Enhanced detection accuracy is observed with increased sensors, resulting in a notable reduction in false detections and overall improved accuracy, surpassing reference fusion-based models. However, despite the value of LiDAR in 3D object detection, its limitations in capturing color information and detailed visual features may render it less optimal for tasks such as traffic sign classification and recognition. Integrating LiDAR with complementary sensor technologies capable of providing color and visual data can augment the quality control capabilities of autonomous vehicles in these specific areas. Their study showed the influence of precipitation and fog on LiDAR performance. The results showed that the retroreflective film was more effective in preserving LiDAR performance under adverse weather conditions. This emphasizes the importance of understanding LiDAR’s efficacy in terms of varying degrees of visibility. This research aims to address the existing gap in assessing the efficacy of LiDAR systems under different levels of visibility. By meticulously evaluating the intensity surrounding traffic signs, this study aims to enrich the knowledge base and ultimately improve the reliability and precision of AV navigation systems for safer and more efficient autonomous driving experiences.

## 3. Methodology

This section delineates the methodological framework employed in the research, comprising two primary components: handheld retroreflectometer measurements and analysis grounded in the intensity extracted from AV LiDAR assessments of traffic sign visibility. Furthermore, the research uses Python programming for data analysis to discern the significance of variables influencing road signs’ retroreflectivity. Following this, the collected data undergoes thorough comparison and statistical analysis. The integration of these methodologies affords a holistic assessment of traffic sign visibility, fostering a cohesive and collaborative approach to evaluation within the study.

### 3.1. Data Collection

The data collection process involved handheld retroreflectometers and two different AVs equipped with sensors. The study tested 160 signs in two distinct urban areas within Gyor City, Hungary. One of the sites was the university campus area, which mainly consisted of low-volume roads and traffic signs at least ten years old. The other site was a roundabout area with two high-volume roads near the city limits, and the traffic signs there were only 2 to 3 years old. The sampled signs included a variety of sheet classes, ranging from old to new, and displayed different positions, meanings, and colors. The survey did not include overhead signs. This comprehensive approach ensured a robust dataset that accurately represented the nuanced characteristics of traffic signs in urban environments.

#### 3.1.1. Handheld Retroreflectometer

The RetroSign GRX 554 retroreflectometer, manufactured by DELTA—a part of FORCE Technology (Hørsholm, Denmark) was used as the primary instrument for all measurements in this study. This device follows the European standard EN 12899-1 [22] to determine the coefficient of retroreflection. Measurements were taken under an illumination angle of 5° and observation angles of 0.2°, 0.33°, and 1°. Each measurement involved additional data such as sample color, ambient temperature, relative humidity, and GPS coordinates.

**Figure 1:**
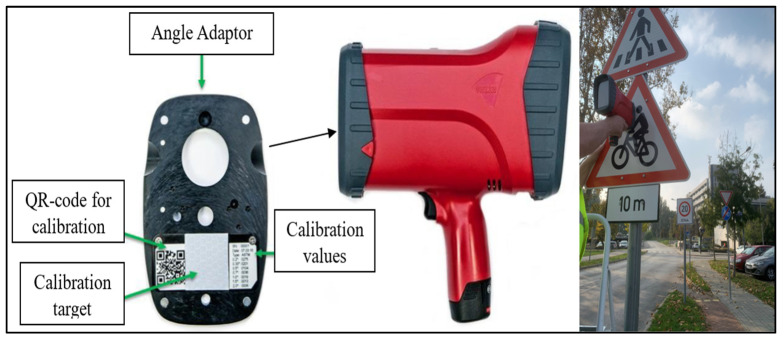
Calibration and data gathering using a handheld retroreflectometer.

**Figure 2:**
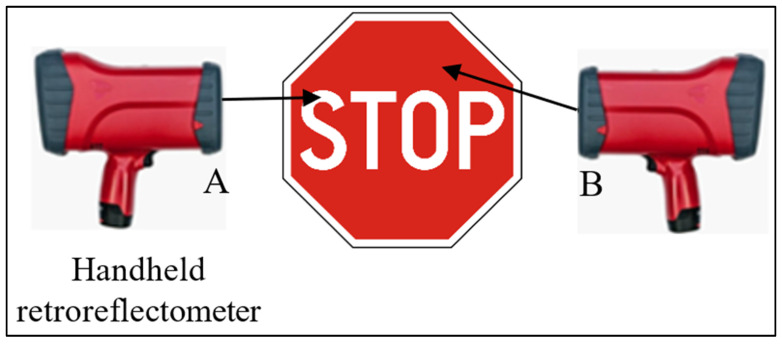
Retroreflective coefficient measurement of traffic signs. (**A**) Legend and (**B**) background.

The RA values were obtained through a calibration process. The device was first calibrated by attaching the calibration standard to the designated “calibration side”. Then, the front plate was attached to the “measuring side”. Direct measurements were taken by placing the device perpendicular to the surface of the traffic sign and initiating the measurement process. The instrument provided measurement values for each observation. Additionally, the handheld retroreflectometer allowed four readings for each sign color, including the background and legend components. A consistent measuring principle was maintained throughout all measurements. Figure 1 visually explains the device calibration and field data collection procedures, while Figure 2 illustrates the process for measuring the traffic sign’s face color. Finally, the data were efficiently transferred to a computer in Excel format.

Retroreflective characteristics data for 160 in-service traffic signs were systematically gathered using a handheld retroreflectometer. From these, 112 were RA1 type, while we had 48 signs of RA2 type.

#### 3.1.2. LiDAR Data

In this study, two cars equipped with sensors belonging to the Vehicle Industry Research Center at Széchenyi István University were used:

1. Nissan Leaf

**Figure 3:**
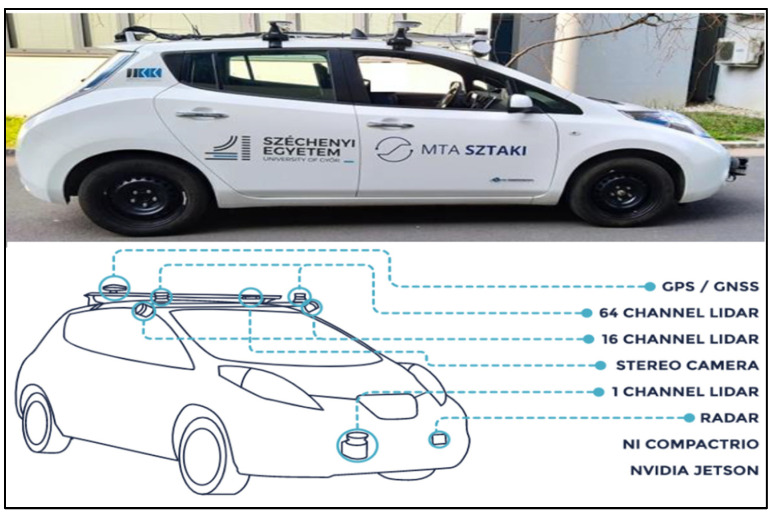
Nissan Leaf vehicle fitted with sensors [23].

The Nissan Leaf was equipped with four LiDAR sensors and two GPS receivers mounted on its roof for georeferencing. A stereo camera embedded in the vehicle created a detailed video log and supplemented frame information obtained from the LiDAR scanner, giving a comprehensive view of the surroundings. The car featured two Ouster Optical Sensor LiDAR (OS1) 64-channel sensors positioned on the roof at a 0° orientation angle, covering a wide angular range. Additionally, two Velodyne VLP16 Puck 16-channel sensors were strategically mounted at 60° and −60° orientations on the car’s upper right and left edges. These additional sensors contributed to the intensity of the point cloud, improving object identification capabilities, as shown in Figure 3.

It is worth noting that all four LiDAR sensors were active during the data collection process. However, the decision was made to extract data from the left and right 64-channel LiDAR sensors, denoted as (2LRLiDAR). This selective extraction was based on the large amount of data collected by these primary sensors and the recognition that the Velodyne 16-channel sensors, with their 60° and −60° orientations, often struggled to identify vertical traffic signs. This strategic approach ensured focused and efficient data acquisition, optimizing the dataset for subsequent traffic sign visibility and retroreflectivity analysis.

2. Lexus RX450h

**Figure 4:**
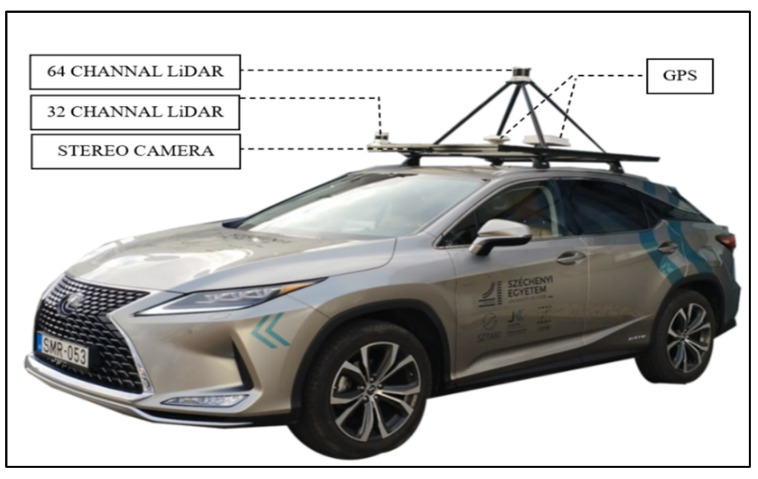
Lexus RX450h vehicle fitted with sensors, adapted from [24].

The Lexus RX450h had three LiDAR sensors and two GPS receivers mounted on its roof for georeferencing purposes. Like the configuration in the Nissan Leaf, a stereo camera embedded in the vehicle generated a comprehensive video log and supplemented frame information from the LiDAR scanner. The vehicle featured one Ouster Optical Sensor LiDAR (OS2) 64-channel sensor positioned at the car’s center, covering a wide angular range. Two Ouster Optical Sensor LiDAR (OS1) 32-channel sensors were mounted on the car’s upper right and left top. All three LiDAR sensors were positioned at a 0° orientation angle, as depicted in Figure 4. Considering the substantial volume of data collected by this primary sensor, only the central 64-channel LiDAR sensor, referred to as (1CLiDAR), was activated during the data collection process. This selective activation ensured focused and efficient data acquisition, allowing the vehicle the capacity to capture detailed information necessary for subsequent traffic sign visibility and retroreflectivity analyses.

3. Difference between 2LRLiDAR and 1CLiDAR Systems:

The primary distinction between the 2LRLiDAR system used in the Nissan Leaf and the 1CLiDAR system used in the Lexus RX450h lies in their data acquisition strategies. The 2LRLiDAR system utilizes both left and right 64-channel LiDAR sensors, offering a broader perspective of the surroundings, particularly aiding in identifying vertical traffic signs. Conversely, the 1CLiDAR system focuses uniquely on the central 64-channel LiDAR sensor, enabling streamlined data acquisition but potentially sacrificing peripheral information. These differences in data acquisition strategies have significant implications for subsequent analyses, as discussed in detail within the results section.

### 3.2. Data Extraction and Preparation

The LiDAR sensors on both vehicles were configured differently, and the data extraction methods employed distinct approaches. For 2LRLiDAR, data recording utilized Rosbag1 files, while 1CLiDAR applied the updated Rosbag2 file format. The point cloud data, which contains the crucial attribute of intensity or return signal strength, played a pivotal role in the analysis, particularly in estimating traffic sign retroreflectivity. The extraction process of 2LRLiDAR point cloud intensity involved a series of steps using various software packages, outlined as follows.

**Figure 5:**
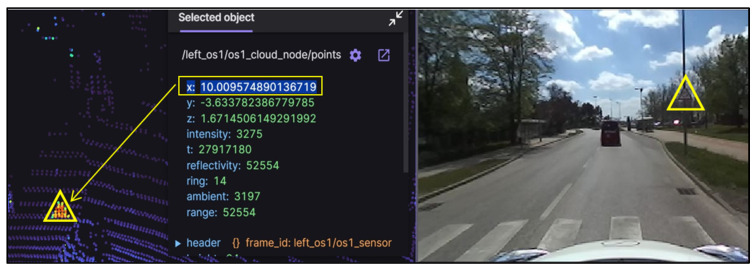
Distance from LiDAR to traffic sign by Foxglove Studio.

In the scanning process, 60 traffic signs were recorded. Despite the inherent limitation of LiDAR sensors in supporting the export of raw data into standard (.las or .laz) format output files, a custom Python script was developed to facilitate the conversion of raw data into point cloud data (.pcd) format. This generated 1874 files per LiDAR sensor, each containing 0.05 s of data acquisition. The Foxglove Studio software, version 2.4.0, was pivotal in precisely determining the time and vehicle position, which was crucial for identifying the frame that captured the sign within the camera’s view at a specified distance of 10 m, as illustrated in Figure 5.

The study utilized CloudCompare 2.6.3, a well-known open-source software specialized in 3D point cloud and mesh processing, to manage the identified framework for analyzing and extracting data from the point cloud file. This process involved several steps in enhancing the visibility of traffic signs and selecting relevant data points while excluding extraneous ones from the dataset. The first step was to increase the number of cloud points by a factor of 5. This was carried out to improve the visibility of traffic signs and facilitate accurate manual selection. Extraneous points were excluded from the dataset.

**Figure 6:**
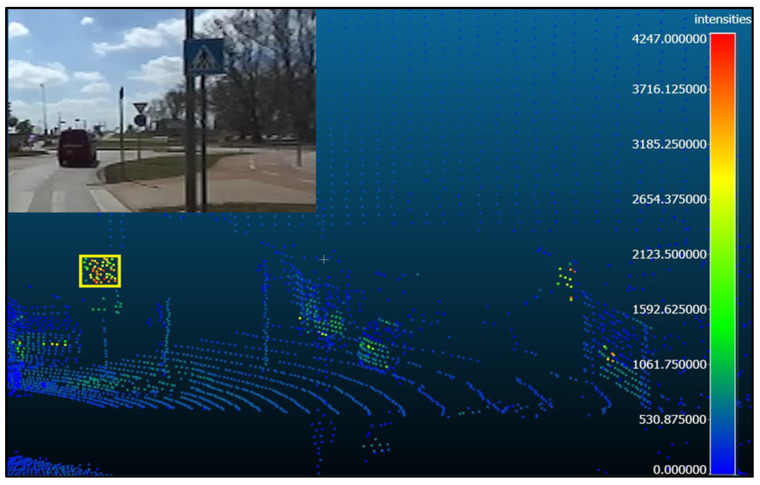
Illustrative example demonstrating LiDAR intensity extraction.

Figure 6 demonstrates the data extraction process from the point cloud file using CloudCompare. This illustrative graphical representation elucidates the meticulous methodology for extracting and analyzing intensity readings from the specified area within the point cloud. This process was crucial in accurately identifying and analyzing traffic signs in the dataset.

**Figure 7:**
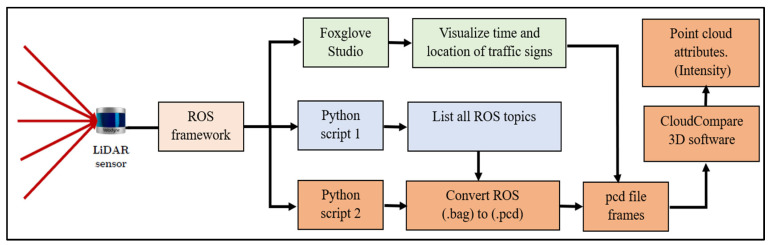
Summary of the software framework for data acquisition.

Furthermore, Figure 7 visually represents the intricate framework utilized for data processing. This graphical illustration comprehensively visualizes the sequential stages encompassing the amplification, selection, and analysis procedures applied to the point cloud data. It emphasizes the systematic approach adopted in data refinement, showcasing the thoroughness and precision employed in extracting and analyzing intensity readings for subsequent assessments of traffic sign visibility and retroreflectivity.

The data extracted from the 2LRLiDAR, which cover 60 signs, have been classified according to specific criteria. It is important to note that all data collected from the 2LRLiDAR were first measured using a handheld retroreflectometer. This approach ensures a thorough and multi-faceted dataset, incorporating LiDAR-derived intensity readings and retroreflectivity measurements.

To extract point cloud intensity from 1CLiDAR, the process relied on the average intensity of each traffic sign, which was obtained using Foxglove Studio. The traffic signs were manually selected from the camera panel, and 20 points were chosen for each sign from the 3D point panel. The average intensity of these points was then calculated. This process was repeated for a total of 105 traffic signs. The extracted data, which include the average point cloud intensity for each sign, were then classified based on the established criteria.

## 4. Data Analysis and Result Discussion

The following analysis reveals some of the the nuances of the relationship between LiDAR point cloud data and handheld retroreflectivity coefficients, highlighting potential applications and limitations. The section concludes with discussions on the significance of the findings, offering informed recommendations and suggesting future research directions in traffic sign retroreflectivity assessment.

### 4.1. Handheld Retroreflectivity Coefficients

This retroreflectivity analysis investigates 160 traffic signs, comprising 111 classified under RA1 and 49 under RA2. This study delves into the intricate details of retroreflective coefficients associated with these distinct sign classes, aiming to provide valuable insights into the visibility and compliance of traffic signs on roadways. By scrutinizing this extensive dataset, we seek to contribute to understanding retroreflectivity performance, offering implications for road safety standards and optimizing signage for enhanced visibility and communication on road networks.

#### 4.1.1. Criteria

The data analysis developed in multiple stages, applying specific criteria to assess the retroreflective coefficients of all traffic signs. The measurements were then carefully compared with the standards outlined for Class RA1 and RA2, as specified in the European standard EN 12899-1. This standard, emphasizing a minimum retroreflective coefficient value, should not fall below 70%, except for the white color, as indicated in Table 1 [22]. Strict adherence to this established standard ensures the rigor and reliability of our assessment of traffic sign visibility, aligning our research with recognized industry criteria.

The analysis revealed significant trends when evaluating the retroreflective coefficients of the 111 RA1 traffic signs and 49 RA2 signs according to the European standard EN 12899-1. Within the RA1 category, 54.1% (60 signs) met the required standards, demonstrating a commendable adherence to reflective performance criteria. However, a considerable proportion of RA1 signs (45.9% or 51) fell outside the standard ranges. This observation highlights the need for a thorough examination to identify potential factors contributing to this deviation and implement corrective measures. On the other hand, the RA2 signs showed a higher compliance rate, with 87.8% (43 signs) meeting the specified standards. However, 12.2% (6 signs) of RA2 signs displayed retroreflective coefficients beyond the acceptable ranges, indicating the need for a focused investigation into sources of non-compliance. The comprehensive evaluation of 160 signs, encompassing both RA1 and RA2 categories, emphasizes the importance of thoroughly assessing manufacturing and maintenance processes. This scrutiny is crucial to adhere to the EN 12899-1 standard consistently. It forms a critical basis for ongoing quality improvement initiatives within traffic sign materials and reflective performance (see Table 2 for detailed results).

Based on the visual scape, Table 3 lists non-conforming traffic signs in the RA1 and RA2 categories, differentiating between the background and legend and integrating both aspects. In the RA1 category, out of the 51 signs falling outside the standard ranges, a substantial majority (66.7%) pertain to non-compliance in the legend, followed by 29.4% in the background and a minor 3.9% in both elements. This disparity underscores the potential challenges related to the text or symbolic elements impacting retroreflective performance. Conversely, in the RA2 category, where there are only six instances of non-conforming signs, all instances (100%) are attributed to issues with the legend. The absence of non-conformities in the background for RA2 signs highlights a distinct pattern, potentially pointing to specific challenges related to text legibility or material characteristics. This nuanced analysis provides valuable insights for targeted interventions in sign design and production processes to enhance retroreflective performance, particularly in the legend element.

Table 4 analyzes the non-conformance of retroreflective coefficients within the RA1 and RA2 traffic sign categories, organized by sign color. Among RA1 signs, white signs have the highest non-conformance rate at 43.3%, followed by red signs at 7.8%. In contrast, blue signs have a relatively low non-conformance rate of 6.3%. The high non-conformance rate for white and red signs suggests potential challenges in achieving retroreflective standards for these colors. Interestingly, gray, green, and yellow RA1 signs consistently meet standards, indicating a strong reflective performance in these colors. In the RA2 category, all sign colors conform to standards, indicating a positive trend in reflective efficiency.

#### 4.1.2. Reflectivity Distribution

**Figure 8:**
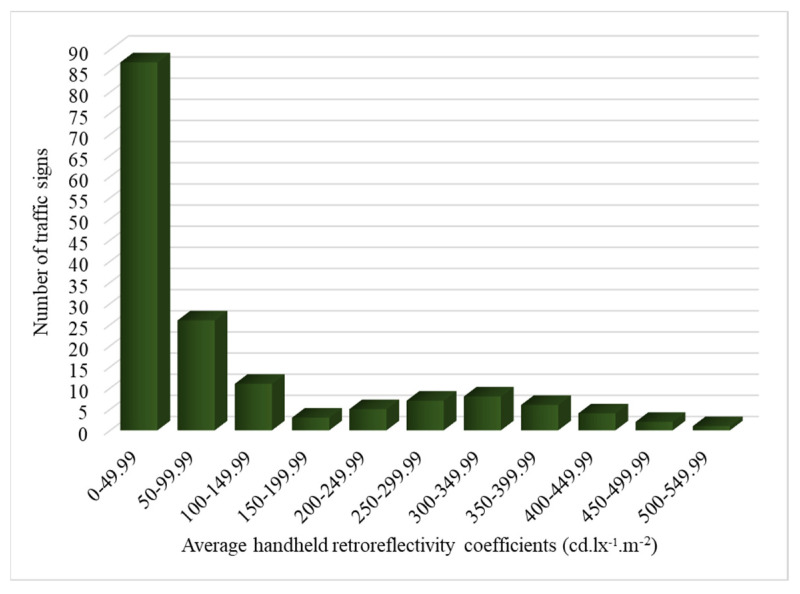
Retroreflective coefficient distribution in collected dataset signs.

Figure 8 provides the distribution of reflective performance within the measured range. The distribution across the specified coefficient groups showcases a predominantly higher concentration within the lower ranges, with 87 signs falling within the 0–49.99 range. However, as the coefficients increase, the frequency diminishes, demonstrating a decreasing trend in retroreflective efficiency. This observation suggests a potential variability in the retroreflective properties of the examined signs, emphasizing the need for an examination of manufacturing processes and materials to ensure uniform adherence to visibility standards.

The retroreflective coefficients measured for the legend and background elements of traffic signs, as shown in Table 5, provide insights into the reflective properties of these signs. The findings indicate considerable variability in retroreflective performance among the legend and background elements, with relative standard deviations well over 100%. This reflects significant fluctuations in retroreflectivity within the sample. The average retroreflective coefficient between the legend and background elements is 106, with a standard deviation of 126 and a relative standard deviation of 119%. While this average represents the traffic signs’ overall reflective performance, the sample’s notable variability highlights the complexity and variability inherent in retroreflective properties.

An independent two-sample *t*-test was conducted to examine whether there is a significant difference in mean retroreflectivity between the legend and background. The analysis was performed using the Statistical Package for the Social Sciences (SPSS) software, version 27.0.1, and the results are presented in Table 6. With a significance level (α) of 0.05, a two-tailed test, and 318 degrees of freedom (df), the critical value (CV) from the Student’s t-distribution was calculated to be 1.9675. The calculated t-value (t-statistic = 1.872) is lower than the critical value (1.9675), indicating no statistically significant difference. The *p*-value (0.06) exceeds the chosen significance level (α = 0.05). Consequently, the 95% confidence interval for the difference in means includes zero, providing strong evidence to accept the null hypothesis and conclude that there is no noticeable difference in the average retroreflectivity between the legend and background. Therefore, the claim that there is no difference in means is supported. However, based on the data, it can be inferred that the retroreflectivity values for the legend and background are similar. Notably, most backgrounds (57.5%) were blue, so further research is needed to investigate the potential impact of background color on retroreflectivity levels.

**Figure 9:**
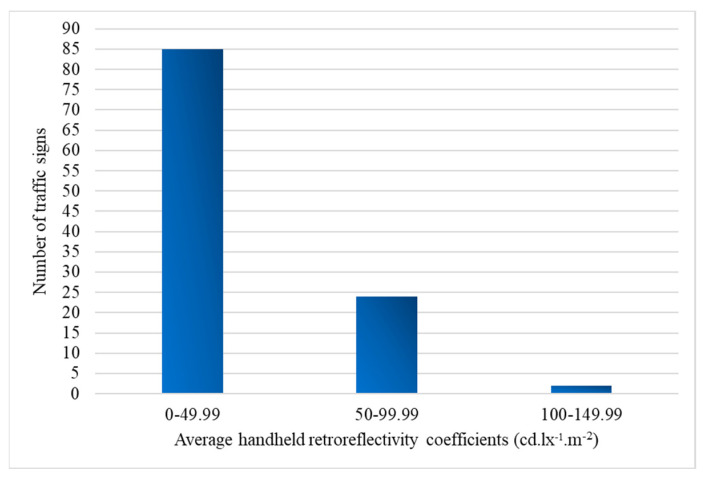
RA1 signs reflectivity profile.

Figure 9 focuses on RA1 traffic signs and shows a distinct pattern. Most signs (85 in total) have retroreflective coefficients falling within the 0–49.99 range, consistent with the overall trend observed in all signs. However, 24 RA1 signs deviate from this trend, with coefficients falling in the 50–99.99 range.

**Figure 10:**
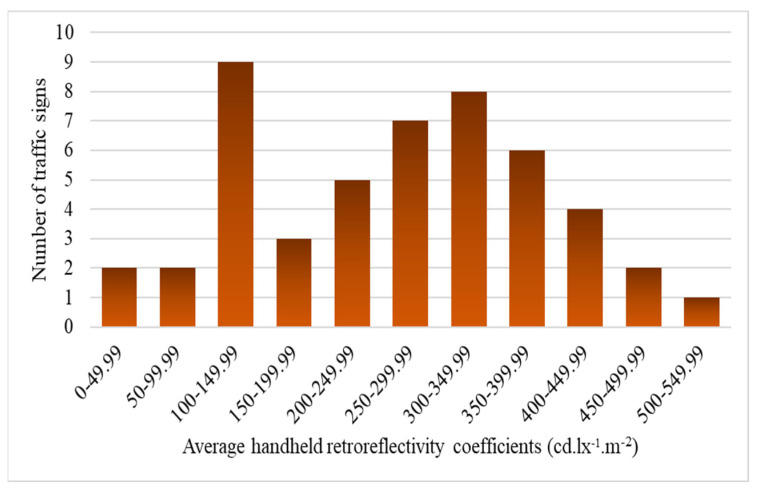
Reflectivity patterns in RA2 Signs.

Figure 10, dedicated to RA2 traffic signs, shows a more widely distributed pattern across various retroreflective coefficient ranges. RA2 signs demonstrate a more balanced representation in lower and higher coefficient groups, with a notable concentration in the mid-range categories. RA2 signs in the higher coefficient ranges suggest a consistent reflective performance within this class.

**Figure 11:**
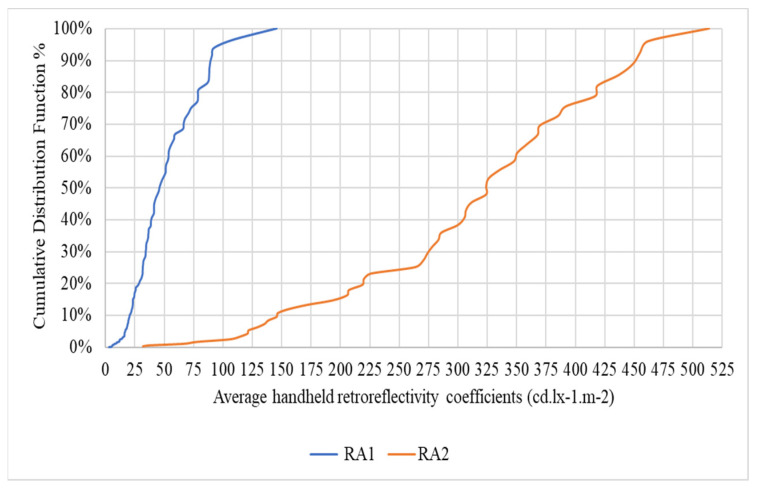
Cumulative Distribution Function (RA1 and RA2).

The Cumulative Distribution Function (CDF) analysis for RA1 and RA2 traffic signs provides a dynamic perspective on the distribution of retroreflective coefficients within these distinct categories, as shown in Figure 11. In the case of RA1 signs, the CDF reveals a gradual increase, with the majority (85%) exhibiting coefficients within the range of 0–49.99. This suggests a consistent prevalence of lower retroreflective coefficients within the RA1 class. On the other hand, the CDF for RA2 signs illustrates a more balanced distribution, reflecting a broader range of coefficients. Notably, 81.6% of RA2 signs exhibit coefficients within the range of 0–99.99, emphasizing the significance of mid-range reflective performance within this category.

**Figure 12:**
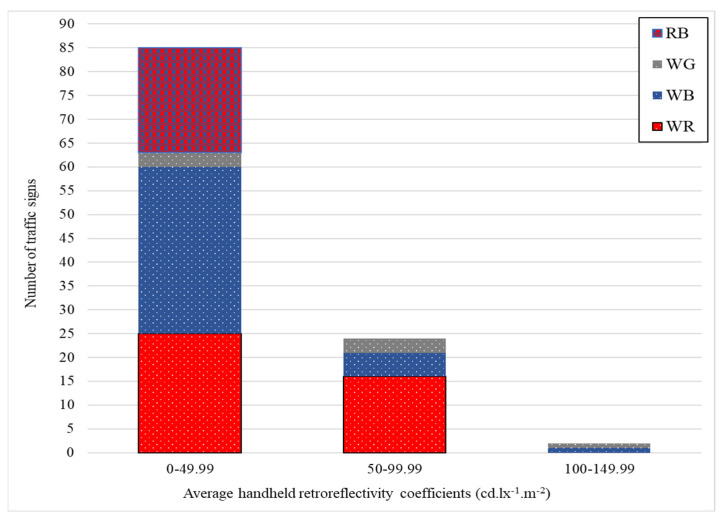
Retroreflective coefficient distribution in RA1 signs by color groups.

**Figure 13:**
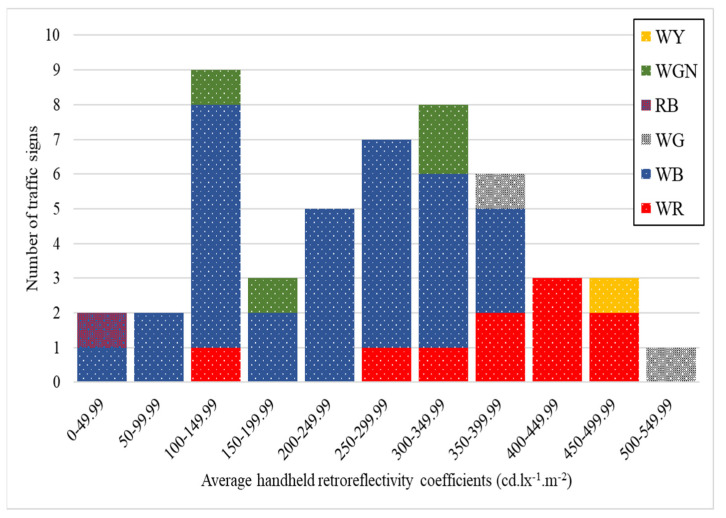
Retroreflective coefficient distribution in RA2 signs by color groups.

Figure 12 and Figure 13 visually represent the distribution of retroreflective coefficients in RA1 and RA2 signs across various color groups. Each color group is further segmented based on specified coefficient ranges. The distinctive colors, namely WR (white and red), WB (white and blue), WG (white and gray), RB (red and blue), WGN (white and green), and WY (white and yellow), offer a clear visual representation of the retroreflective performance within each color category. These illustrations are a valuable tool for assessing variations in retroreflective coefficients across different color combinations, enabling a quick and comprehensive understanding of the reflective characteristics within the RA1 and RA2 signs classification.

Table 7 and Table 8 analyze the retroreflective coefficients for RA1 and RA2 traffic signs, including their legends and background elements. The findings indicate considerable variability in retroreflective performance among legends, with a relative standard deviation of 80%, and backgrounds, with a relative standard deviation of 97%, within the RA1 category. In comparison, for RA2 signs, the mean retroreflective coefficient for legends is notably higher at 306, with a standard deviation of 158.8 and a relative standard deviation of 52%. Similarly, the mean retroreflective coefficient for backgrounds is 221, with a standard deviation of 221.9 and a relative standard deviation of 101%. The average retroreflective coefficient between legend and background elements for RA2 signs is 263, with a standard deviation of 123.6 and a relative standard deviation of 47%. These results underscore a significant increase in retroreflectivity for RA2 signs compared to RA1 signs, with retroreflectivity data for RA2 being two to four times higher or even more.

The reflective characteristics of RA1 and RA2 were analyzed to explore potential differences in their mean reflectivity levels. Using a two-sample *t*-test, a t-statistic of −18.49 (where the negative sign denotes a lower mean) was obtained, surpassing the critical value of 1.9751, with a *p*-value approaching zero, lower than the significance level (α = 0.05). Additionally, the 95% confidence interval for the difference in means excludes zero, leading to the rejection of the null hypothesis. Therefore, there is a significant difference in mean reflectivity between RA1 and RA2. Specifically, RA2 signs display, on average, a significantly higher retroreflectivity compared to RA1 signs. This observation highlights the crucial role of retroreflective properties in selecting and maintaining traffic signs to ensure optimal visibility and safety. The authorities responsible for traffic sign management should consider the superior retroreflective attributes of RA2 signs for enhanced visibility, especially under low-light conditions. Furthermore, implementing regular monitoring and maintenance initiatives is essential to maintain the effectiveness of retroreflective materials on traffic signs over time, thereby improving road safety for both motorists and pedestrians.

Subsequent *t*-tests were conducted on the legend and background retroreflectivity of RA1 and RA2 to investigate possible variations. For RA1, the analysis produced a t-statistic of 1.94 with a corresponding *p*-value of 0.054, while for RA2, the t-statistic was 2.18 with a *p*-value of 0.032, both under α = 0.05. In the case of RA1, the observed *p*-value surpassed the predetermined threshold, indicating an absence of statistically significant difference in mean retroreflectivity between the legend and background. Conversely, for RA2, the computed *p*-value fell below the threshold, signifying a notable difference in mean retroreflectivity between the legend and background. These findings suggest potential differences in retroreflective performance between different legends and backgrounds, necessitating further investigation to clarify underlying factors influencing retroreflectivity levels.

Furthermore, the analysis reveals significant differences in retroreflectivity between colors, with RA2 signs exhibiting notably higher retroreflective coefficients than RA1 signs. Additionally, the susceptibility of retroreflectivity measurements to pointwise variability emphasizes the necessity for meticulous measurement techniques and quality control measures to ensure accurate and consistent assessment of retroreflective properties.

### 4.2. LiDAR Intensity Analysis

This section presents an analysis and discussion concerning the data acquired through LiDAR sensors. Specifically, the investigation examines data gathered from 60 signs scanned by 2LRLiDAR and 105 by 1CLiDAR, forming the basis for subsequent analytical insights and interpretations.

#### 4.2.1. Analysis of 2LRLiDAR Data

Intensity data extracted from 2LRLiDAR provides insights into the distribution of intensity values across various ranges. Among the categorized intensity groups, most signs fall within the 2000–2499 intensity range, comprising 35 signs from the total dataset. This prevalence suggests a commonality in the reflective properties or materials of the assessed signs, resulting in a consistent intensity reading within this range. Conversely, fewer signs are observed in the lower and higher intensity ranges, indicating variability in reflective characteristics among the assessed signs.

**Figure 14:**
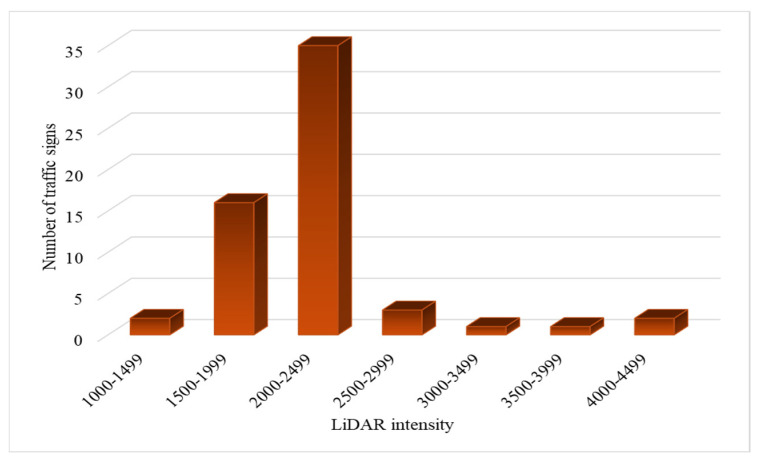
Intensity distribution in traffic signs using 2LRLiDAR.

Figure 14 visually represents the distribution of traffic signs across different intensity ranges. It illustrates the dominance of the 2000–2499 intensity range. A gradual decline follows a notable peak in sign frequency as intensity values deviate from this range. This distribution pattern emphasizes the concentration of signs within a specific intensity range, potentially indicating trends in reflective properties among the assessed signs.

Considering the composition of the sheet class within the dataset is crucial for accurately contextualizing the intensity distribution. Most signs belong to sheet class RA1, so the intensity distribution primarily reflects the characteristics of RA1 signs. However, including RA2 signs, although in smaller numbers (only six signs), introduces variability in the intensity distribution. Notably, the 2000–2499 intensity range remains prominent among RA2 signs, with five signs aligning with the prevalence observed in RA1 signs.

The statistical analysis conducted on the intensity data provides insights into the comparative characteristics of RA1 and RA2 signs, as shown in Table 9. The results indicate minor differences in mean intensity between RA1 and RA2 signs, with RA2 signs demonstrating slightly higher mean intensity and lower variability. However, the relative standard deviation within each group suggests moderate variability in intensity measurements across both RA1 and RA2 sign categories, emphasizing the importance of considering variability in intensity assessments for effective sign design and visibility enhancement.

Based on these values obtained from the independent samples, the *t*-test resulted in a t-statistic of approximately −0.982, which is lower than the critical value of 2.002. The corresponding *p*-value is approximately 0.330. The 95% confidence interval for the difference in means includes zero, indicating no significant difference in mean intensity between RA1 and RA2. Therefore, we cannot reject the null hypothesis, suggesting that the mean intensities of RA1 and RA2 are comparable within the observed data.

**Figure 15:**
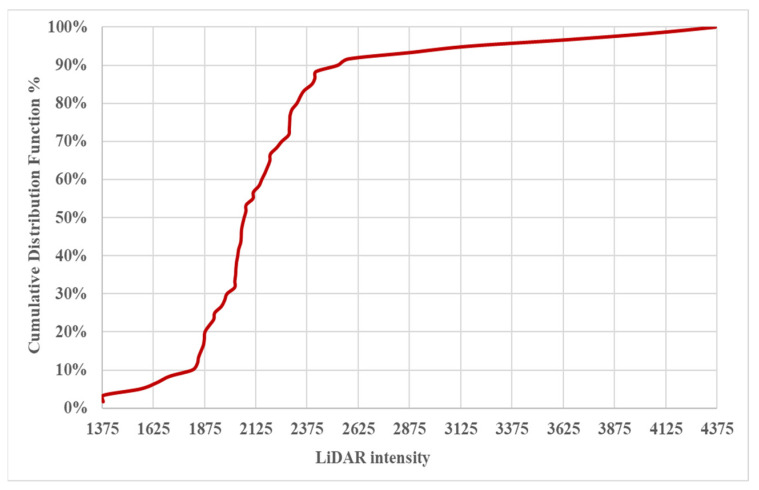
Cumulative Distribution Function for 2LRLiDAR intensity.

Figure 15 displays the Cumulative Distribution Function (CDF) for 2LRLiDAR intensity data collected from the evaluated traffic signs. This curve comprehensively represents the distribution of intensity values within the dataset. As the intensity values increase, the cumulative frequency also increases, indicating the prevalence of certain intensity levels among the evaluated signs. The curve shows a significant rise in cumulative frequency within the 2000–2499 intensity range, suggesting a commonality in reflective properties or material characteristics. Beyond this peak range, the curve gradually flattens, indicating a decrease in the cumulative frequency of signs with higher or lower intensity values. The CDF analysis offers valuable insights into the overall reflective performance of the evaluated traffic signs, aiding in identifying prevalent intensity levels and potential outliers. This information is crucial for informing decision-making processes related to sign maintenance, visibility enhancement, and road safety measures.

Table 10 analyzes intensity measurements for different sign colors, including WR, WB, RB, and signs with white (WSN). The results show a moderate level of variability, with the mean intensity recorded as follows: WB (2260), WR (2129), WG (2233), RB (2154), and WSN (2205). While there are noticeable differences in intensity among the different sign colors, the overall variability remains consistent. The presence of moderate variability in intensity measurements within each color category is supported by the moderate relative standard deviations, which range from 21% to 26% across different color groups. Furthermore, *t*-tests show that the mean intensity values for each color category are not statistically significantly different. These findings suggest that while slight variations in intensity among different sign colors may exist, the overall variability remains stable. Therefore, the impact of sign color on intensity measurements may not be significant, a conclusion supported by references [18,21]. Further investigation into other factors that may affect sign visibility and effectiveness is warranted.

#### 4.2.2. Analysis of 1CLiDAR Data

Analyzing intensity data extracted from 1CLiDAR for 105 traffic signs provides valuable insights into the distribution of intensity values across various ranges. Among the categorized LiDAR intensity groups, most signs fall within the 1650–1699 intensity range, with 73 signs accounting for a significant portion of the dataset. This concentration suggests a prevalent intensity level among the assessed signs, potentially indicating standard materials or surface characteristics that influence LiDAR intensity readings. Conversely, fewer signs are observed in the lower intensity ranges, suggesting a lower prevalence of lower-intensity reflective properties among the assessed signs.

**Figure 16:**
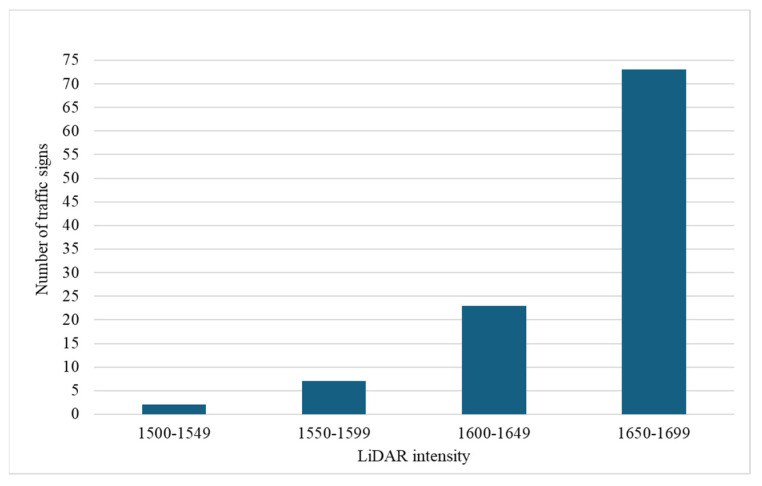
Intensity distribution in traffic signs using 1CLiDAR.

Figure 16 visually represents the distribution of traffic signs across different LiDAR intensity groups, clearly depicting intensity distribution trends. The chart illustrates the dominance of the 1650–1699 intensity range, with a pronounced peak in sign frequency, followed by a gradual decline in sign count as intensity values deviate from this range. This distribution pattern highlights the concentration of signs within a specific intensity range, underscoring potential trends in reflective properties among the assessed signs.

Additionally, the inclusion of sheet classes in the dataset enhances the analysis. Out of the 105 signs, 69 are classified as RA1 and 36 as RA2. Interestingly, most of RA2 signs (33 out of 36) fall within the upper-intensity range of 1650–1699. This indicates that RA2 signs have a higher potential for intensity recovery compared to RA1 signs when evaluated using 1CLiDAR technology. This finding suggests that the 1CLiDAR system has reached its maximum capacity for intensity recovery in the upper range for RA2 signs. This could be due to their ability to reflect more light and provide better visibility, highlighting their potential to improve road safety.

The statistical analysis conducted using 1CLiDAR for traffic sign intensity has demonstrated its effectiveness in providing consistent and precise measurements, as shown in Table 11. The data presented reflect the tool’s ability to accurately measure intensity, with mean values of 1639 and 1662 for RA1 and RA2, respectively, and a narrow standard deviation of 2.0% and 0.5%, respectively. This analysis indicates minimal variability in intensity measurements within both RA1 and RA2 sign categories, highlighting the precision and reliability of 1CLiDAR’s data output. The narrow relative standard deviations within groups, ranging from 0.5% to 2%, further emphasize the tool’s usefulness in assessing and monitoring traffic sign visibility and effectiveness, demonstrating its potential to improve road safety measures. These results collectively suggest a consistent and stable intensity measurement across RA1 and RA2 sign categories when using 1CLiDAR.

The *t*-test results indicate a significant difference in mean intensity between RA1 and RA2 1CLiDAR measurements. The calculated t-statistic of −4.01 is higher than its critical value of 1.98, and the corresponding *p*-value of 0.0001 supports rejecting the null hypothesis. This suggests that the intensity values captured by 1CLiDAR for RA1 and RA2 are not statistically equivalent. Further investigation may be warranted to explore the underlying factors contributing to this difference and their implications for the data analysis or research objectives.

Furthermore, the intensity analysis conducted using 1CLiDAR reveals that the average intensity of traffic signs remains consistent regardless of their color, as shown in Table 12. This analysis demonstrates minimal variation in intensity measurements across different sign colors, with a narrow relative standard deviation of 1.8%. These findings also indicate minimal differences among color categories, highlighting the reliability and consistency of 1CLiDAR in providing precise and accurate intensity data for evaluating the visibility and effectiveness of traffic signs, regardless of their color composition. Overall, these results emphasize the robustness of 1CLiDAR in capturing accurate intensity data, making it a valuable tool for assessing traffic sign visibility and promoting road safety; this conclusion is supported by reference [21].

**Figure 17:**
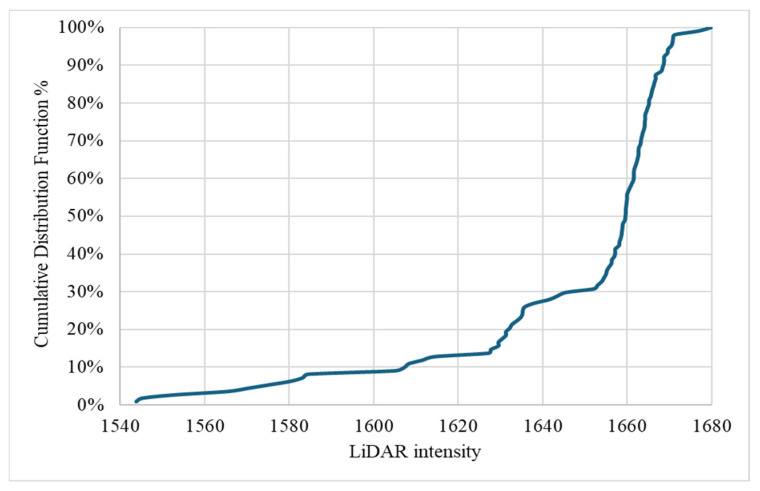
Cumulative Distribution Function for 1CLiDAR intensity.

The CDF analysis conducted for 1CLiDAR intensity data, as illustrated in Figure 17, reveals a significant trend emerging as intensity values approach 1650 and beyond. This trend is depicted by a pronounced upward trajectory in the cumulative curve around the 1650 intensity threshold, continuing to ascend as intensity values increase. This pattern indicates a notable concentration of traffic signs exhibiting higher intensity readings within this upper range, suggesting a prevalence of intense reflective properties among the assessed signs. This behavior underscores the efficacy of 1CLiDAR technology in accurately capturing and quantifying high-intensity reflective properties, which are crucial for ensuring optimal visibility and road safety. By identifying this prominent trend in the Cumulative Distribution Function, valuable insights can be gained into the reflective performance of traffic signs assessed using 1CLiDAR. This information can inform strategies for sign maintenance, visibility enhancement, and overall roadway safety measures. Additionally, the analysis identifies prevalent intensity levels and potential outliers within the dataset, contributing to informed decision-making processes regarding road infrastructure management and safety enhancement initiatives.

### 4.3. Comparison between 2LRLiDAR and 1CLiDAR

**Figure 18:**
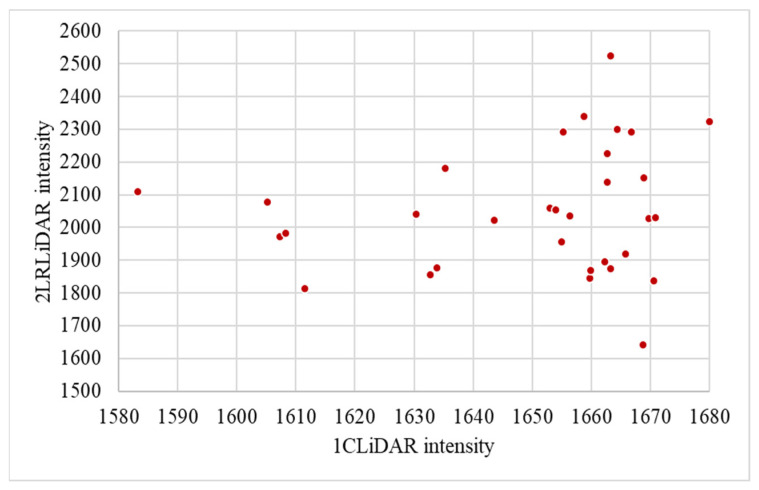
Comparing two LiDAR intensities for the same traffic signs.

The comparison between two LiDAR systems, 2LRLiDAR and 1CLiDAR, reveals distinct characteristics in their intensity measurements. As shown in Figure 18, the scatter plot illustrates the intensity values obtained from both LiDAR systems for the same set of traffic signs. Visually, it is clear that the intensity values from 2LRLiDAR tend to be higher compared to those from 1CLiDAR, as evidenced by the data points appearing higher on the y-axis. This difference suggests that 2LRLiDAR may provide higher resolution or sensitivity in intensity detection, resulting in more pronounced values for the same traffic signs. Notably, augmenting the number of LiDARs results in increased intensity and density, an inference supported by reference [21]. Additionally, the scatter across the x-axis indicates variations in intensity readings between the two LiDAR systems for the 32 traffic signs observed by both LiDARs. These variations could be attributed to differences in LiDAR technology, calibration methods, hardware, processing algorithms, or environmental conditions during data acquisition. Furthermore, ROS1 for 2LRLiDAR data and ROS2 for 1CLiDAR data could also contribute to data quality and accuracy variations due to differences in the underlying software infrastructure.

**Figure 19:**
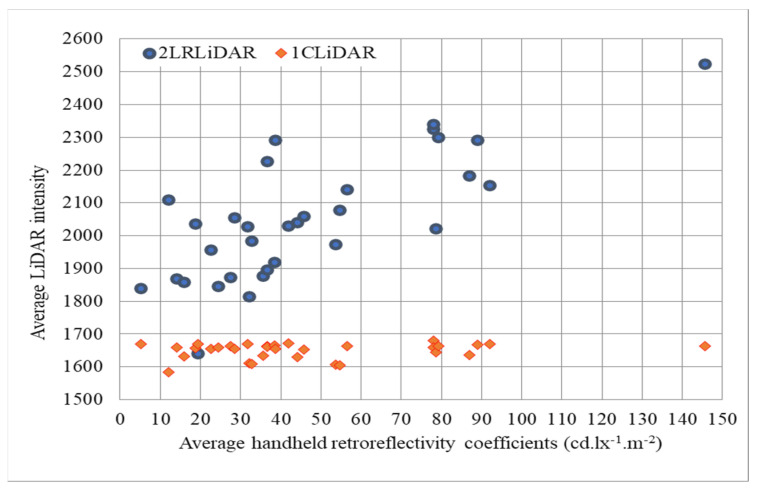
Comparing LiDAR intensities with average retroreflectivity coefficients.

Figure 19 depicts the correlation between the intensity values of the LiDAR systems and the average retroreflectivity coefficients of traffic signs. The x-axis represents the retroreflectivity coefficients arranged from smallest to largest, while the y-axis represents the intensity values obtained from 2LRLiDAR and 1CLiDAR. As the retroreflectivity coefficients increase along the x-axis, the intensity values of 2LRLiDAR gradually increase, indicating a positive correlation between the two variables. However, the intensity values of 1CLiDAR exhibit a more scattered distribution, suggesting a less consistent relationship with the retroreflectivity coefficients. Additionally, comparing the intensity values of both LiDAR systems to the retroreflectivity coefficients reveals discrepancies between them. While the intensity values of 2LRLiDAR closely align with the retroreflectivity coefficients, those of 1CLiDAR show significant variability and do not demonstrate a clear correlation. This highlights the importance of understanding the limitations and characteristics of different LiDAR systems when interpreting intensity data related to retroreflectivity coefficients.

### 4.4. Comparing Traffic Signs and Surrounding Area Intensity

This section analyzes the LiDAR intensity contrast between traffic signs and their surrounding environments. The methodology involved scanning 60 traffic signs using 2LRLiDAR to extract intensity data. Concurrently, the surrounding area of each sign underwent scanning by removing the sign from the point cloud and selecting an adjacent square area measuring (4 × 4) meters. Subsequently, mean intensity readings for the signs and their surrounding areas were computed and contrasted.

Table 13 presents an overview of the mean intensity readings obtained from traffic signs and their surrounding areas. The study reveals a significant difference in the mean intensity readings between traffic signs and their surrounding areas. Specifically, the traffic signs had a mean intensity of 2192, with a standard deviation of 519.8, indicating moderate variability in intensity. In comparison, the surrounding areas had a mean intensity of 625, with a higher standard deviation of 391.0, indicating a broader range of intensity values. The independent samples *t*-test value of 18.663, much higher than the critical value of 1.98, indicates a significant difference between the intensity distributions of traffic signs and their surrounding areas, with a standard error difference of 83.97. Furthermore, the *p*-value approaching zero, which is lower than the significance level (α = 0.05), and the 95% confidence interval for the difference in means, which does not contain zero, provide strong evidence against the null hypothesis of no difference in mean intensities. This statistical analysis highlights the importance of considering intensity differentials when designing and implementing LiDAR-based traffic sign detection systems. By accounting for the significant disparity in intensity levels between traffic signs and background elements, researchers and practitioners can develop more robust algorithms and strategies for accurate and reliable traffic sign recognition, ultimately enhancing road safety and transportation efficiency.

**Figure 20:**
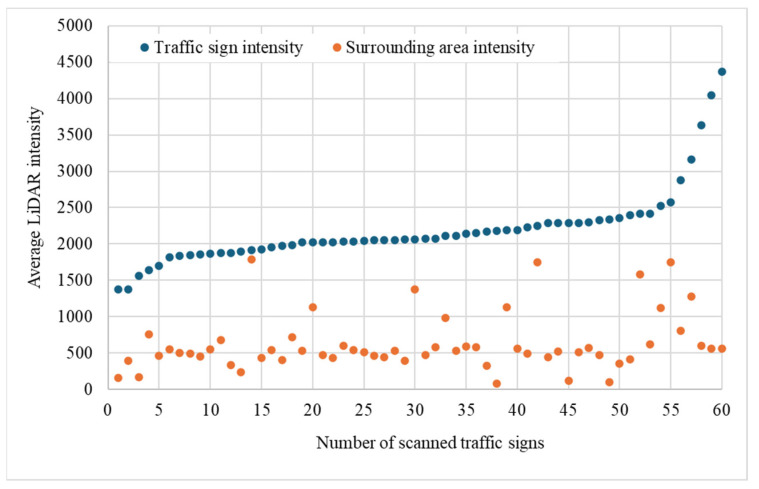
LiDAR intensity analysis of scanned traffic signs and surrounding areas.

Figure 20 clearly shows the relationship between the intensity levels of scanned traffic signs and their corresponding surroundings. The intensity levels of scanned traffic signs range from 1380 to 4366, while the surrounding areas range from 78 to 2876. This stark contrast emphasizes the critical need for precise delineation and characterization of traffic sign features in various environmental conditions.

A thorough understanding of these variations is essential for improving the effectiveness and resilience of LiDAR-based applications in traffic management and urban planning. For example, imagine a traffic sign in a poorly lit area or surrounded by dense foliage. In such cases, the distinct radiometric signatures of the traffic sign and its surrounding environment play a crucial role in determining the accuracy of detection and recognition algorithms. Additionally, the intensity disparity between traffic signs and their surroundings significantly affects LiDAR’s ability to detect and recognize traffic signs accurately. In well-lit areas or clean environments, where there is a significant difference in intensity, LiDAR systems can easily distinguish higher-intensity signals from traffic signs against the lower-intensity background, resulting in improved detection accuracy. However, in poorly lit environments or areas with significant background clutter, where the intensity contrasts between traffic signs and their surroundings are minimal, LiDAR systems may struggle to identify traffic sign features accurately. This limitation can potentially compromise the reliability and effectiveness of traffic sign detection algorithms, leading to safety hazards and disruptions in traffic management systems, especially for AVs.

### 4.5. Relation between Retroreflectivity Coefficients and LiDAR Intensity

The investigation aimed to explore the relationship between retroreflectivity coefficients and LiDAR intensity, aligning with this study’s objectives. To achieve this aim, a thorough correlation analysis was conducted to examine the association between raw intensity data obtained from 2LRLiDAR and 1CLiDAR systems and retroreflectivity coefficients obtained through handheld measurements. This analysis focused specifically on the RA1 and RA2 traffic sign classes and different color compositions to better understand the relationship between these variables.

#### 4.5.1. Relation between Retroreflectivity Coefficients and 2LRLiDAR Intensity

Table 14 shows a limited correlation between intensity, as measured by 2LRLiDAR, and retroreflectivity coefficients. Generally, RA2 signs have higher mean intensities and retroreflectivity coefficients than RA1 signs, suggesting a potential correlation between higher intensity readings and increased retroreflectivity. However, significant variability in retroreflectivity measurements, particularly within RA1 signs, indicates possible inconsistencies in retroreflective performance. Furthermore, there are no significant differences in mean intensity between RA1 and RA2 signs. The analysis also reveals discrepancies in intensity and retroreflectivity measurements between RA1 and RA2 signs, with RA2 signs consistently showing higher values for both parameters. This suggests that RA2 signs may offer better visibility and reflective properties than RA1 signs. However, the significant variability in retroreflectivity measurements across all signs highlights the need for further investigation and the implementation of quality control measures to ensure consistent visibility and reflective characteristics for all traffic signs.

Table 15 displays the analysis of 2LRLiDAR intensity data and retroreflectivity coefficients for signs categorized by the presence or absence of white coloration: signs with white color (WSN) and signs without white color (SNW). The analysis reveals slight variations between the two groups. The mean intensity for WSN is 2205, while SNW shows a slightly lower mean intensity of 2154, indicating a difference of 51 in mean intensity. This suggests that white-colored signs may reflect more light, resulting in slightly higher intensity readings. However, it is noteworthy that the standard and relative standard deviations for intensity are marginally higher for SNW, indicating a wider spread of intensity values within this group. Furthermore, the mean retroreflectivity coefficient for WSN is notably higher at 71 compared to SNW’s mean of 23. This represents a significant difference in retroreflective properties between the two groups. Signs with white coloration generally possess higher retroreflective properties, leading to more efficient light reflection. Understanding these variations is crucial for assessing the effectiveness of traffic signs. Higher intensity readings and retroreflectivity coefficients in WSN suggest that these signs may be more visible and effective in guiding motorists, especially in low-light conditions or adverse weather. Conversely, SNW signs may exhibit greater variability in visibility due to their lower retroreflective properties and slightly lower intensity readings.

**Figure 21:**
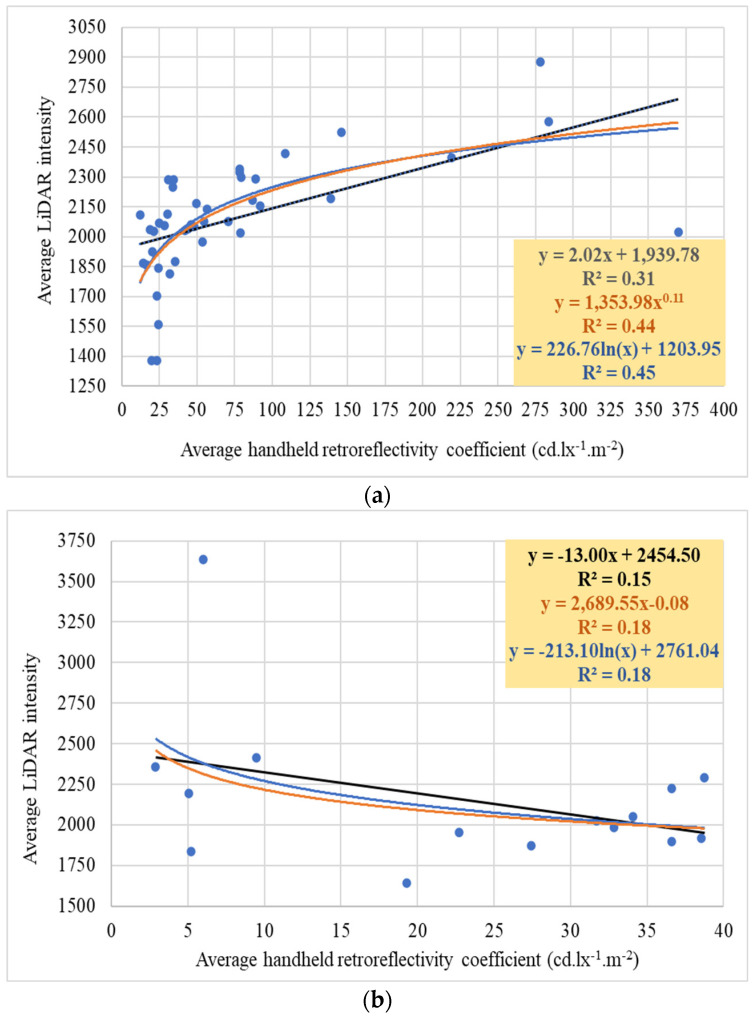

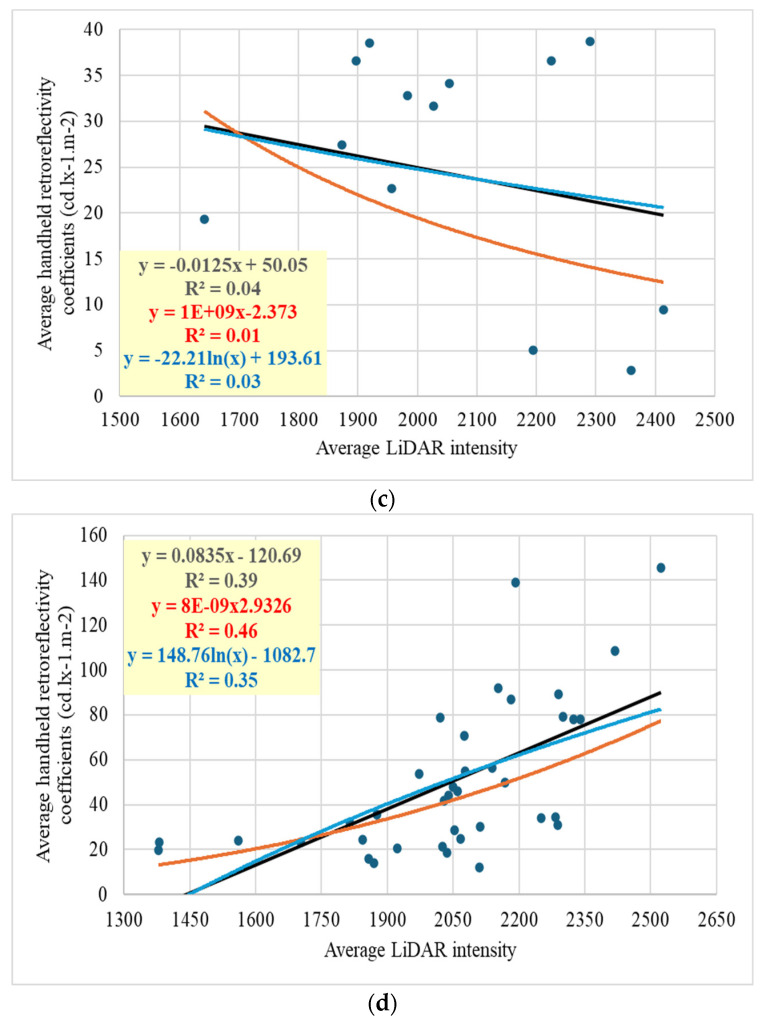
Relations between retroreflectivity coefficient and 2LRLiDAR intensity: (**a**) WSN signs, (**b**) SNW signs, (**c**) WSN signs, and (**d**) SNW signs.

The study’s results suggest a limited association between LiDAR intensity and handheld retroreflectivity coefficients, as illustrated in Figure 21. Using the Modified Z-score method, the graph demonstrates the correlation after excluding outliers. This highlights the constraints caused by inherent differences in measurement methodologies. The conclusion drawn from this analysis emphasizes the urgent need for further investigation and the implementation of quality control measures to understand and standardize intensity and retroreflectivity measurements effectively. While color combinations and materials may influence these metrics, the observed variability within categories suggests that additional factors such as surface condition or manufacturing quality may significantly impact the results. Standardizing these measurements is crucial for improving traffic sign visibility and ensuring road safety. It is also important to note that while LiDARs can detect these variations, they may not always be suitable for quality control.

#### 4.5.2. Relation between Retroreflectivity Coefficients and 1CLiDAR Intensity

Table 16 shows a clear correlation between intensity and retroreflectivity coefficients based on 1CLiDAR data. Signs with higher retroreflectivity coefficients, such as RA2 signs, have higher intensity readings, while those with lower coefficients, like RA1 signs, have lower intensity readings. This suggests that signs with higher retroreflectivity coefficients effectively reflect light back to the source, resulting in higher intensity readings. Additionally, there are statistically significant differences in mean intensity between RA1 and RA2 signs. The analysis also highlights discrepancies in intensity and retroreflectivity measurements between RA1 and RA2 sign classifications. RA2 signs consistently demonstrate superior reflective properties, as shown by their higher intensity readings and retroreflectivity coefficients compared to RA1 signs.

The study reveals a significant difference in the reflective properties of traffic signs based on their coloration. WSN signs have a much higher mean retroreflective coefficient of 179.8 than SNW signs, with a mean intensity of 142.5, as depicted in Table 17. This suggests that the color of a traffic sign can affect its reflective properties. The statistical analysis shows a t-statistic of 4.957, above the critical value of 1.968, and a *p*-value of approximately 0.001, below the significance level. This indicates a significant difference in mean reflectivity between WSN and SNW signs. Additionally, the 95% confidence interval for the difference in means does not include zero, further supporting the conclusion of a significant difference in reflectivity between the two types of signs.

However, it is essential to note that the standard and relative standard deviations for intensity are relatively low for SNW and WSN signs, indicating a consistent distribution of intensity values within each group. The statistical analysis for intensity shows a t-statistic of −1.409, lower than the critical value of 1.983, and a *p*-value of 0.162, above the significance level. This means there is no significant difference in mean reflectivity between WSN and SNW signs. The 95% confidence interval for the difference in means crosses zero, further supporting this conclusion. These findings suggest that WSN signs generally have higher retroreflective properties, resulting in more efficient light reflection. This highlights the importance of considering intensity and retroreflectivity in comprehensive traffic sign visibility and effectiveness assessments. Understanding these reflective properties can inform traffic sign design, placement, and maintenance decisions, ultimately enhancing road safety and traffic management systems.

These findings underline the importance of considering color when optimizing traffic sign visibility and effectiveness, ultimately contributing to enhanced road safety. The 1CLiDAR intensity, ranging from 1600 to 1700 with a relative standard deviation of 1–2%, reflects a narrow range, indicating measurement consistency. However, further investigations are needed to fully understand the relationship between intensity and retroreflectivity coefficients. A deeper understanding of these dynamics can inform targeted interventions to improve signage efficacy and mitigate potential roadway hazards.

**Figure 22:**
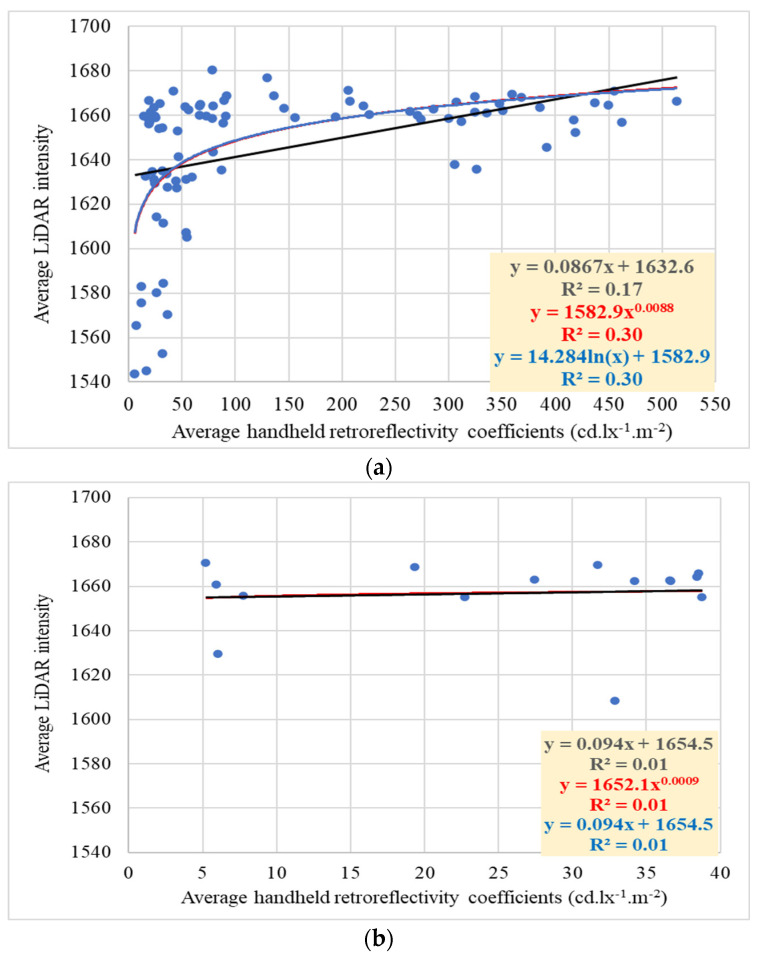

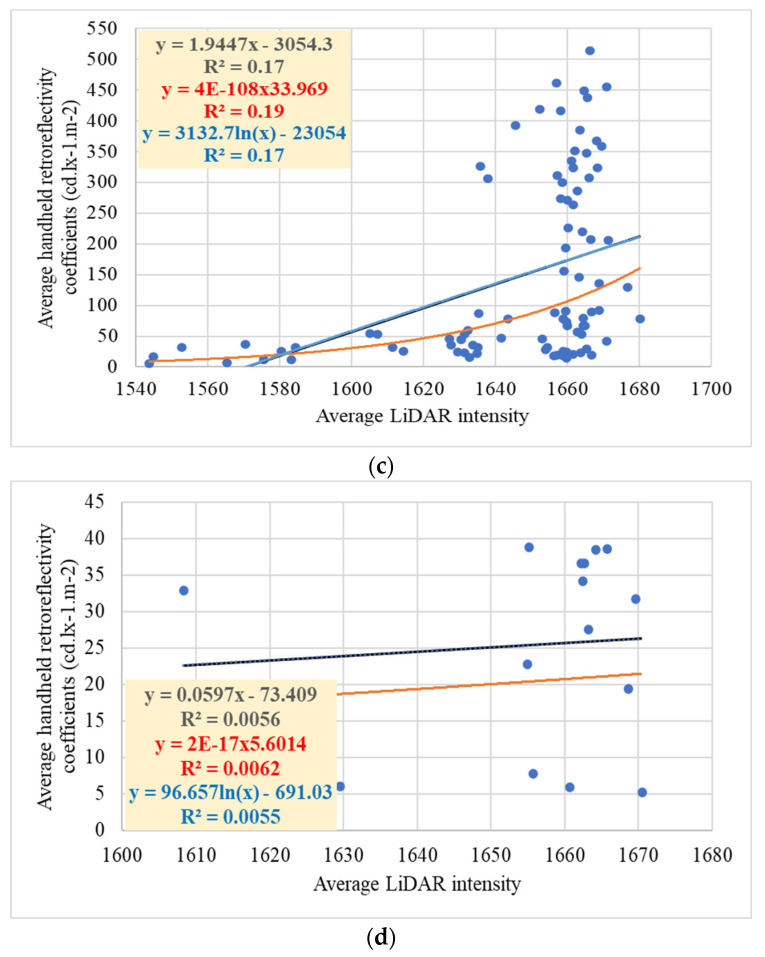
Relations between retroreflectivity coefficient and 1CLiDAR intensity: (**a**) WSN signs, (**b**) SNW signs, (**c**) WSN signs, and (**d**) SNW signs.

The results consistently show a limited correlation between LiDAR intensity and handheld retroreflective coefficients, as shown in Figure 22. These results highlight the limitations of using different measurement methodologies. The observed variation within categories suggests that additional factors contribute to the limitations in correlation, such as differences in measurement processes. Handheld retroreflectometers focus on specific areas of traffic signs to accurately evaluate retroreflectivity, typically measuring a larger area of several centimeters in diameter. While this method ensures precision, it may not fully capture the overall reflectivity properties of the entire sign surface, especially in the presence of surface irregularities and environmental contaminants. In contrast, LiDAR generates a point cloud by emitting laser beams across a broader spatial area, typically measuring a tiny area on a scale of a few millimeters. Challenges arise from inconsistencies in measurement scale, orientation, and surface coverage, which make it difficult to establish a robust relationship.

Additionally, retroreflectivity standards for traffic signs are carefully designed to ensure visibility in various lighting conditions, considering human vision sensitivity, contrast, and environmental factors. Variations in standards by color aim to maintain sign visibility and meaning, ultimately enhancing road safety. In contrast, LiDAR intensity reflects the strength of laser return signals received by sensors, providing insights into material reflectivity, surface characteristics, and object recognition. Unlike retroreflectivity, LiDAR intensity is not standardized and depends on specific applications, with surface color having minimal direct influence. Instead, material reflectivity, surface roughness, and laser beam angle play a more significant role. Operating in the near-infrared range, LiDAR intensity values are complex and not precisely correlated with human-visible colors, highlighting their multifaceted dependence on various factors beyond color alone.

### 4.6. Comparison between 2LRLiDAR Intensity at Different Distances

This analysis examines the intensity data obtained from 2LRLiDAR at varying distances of 10 m, 15 m, and 20 m. This reveals variations attributable to the distance-dependent characteristics of LiDAR sensors and the physical attributes of traffic signs. Table 18 displays the extracted intensity data alongside corresponding averages for each distance. At 10 m, the average intensity registers in 2076, indicating proximity to the traffic sign and yielding higher intensity values due to the amplified signal received by the LiDAR sensor. Moving to a 15 m distance, the average intensity reduces to 1976, approximately 95% of the intensity observed at 10 m. This showcases the robust performance of the LiDAR sensor in maintaining signal strength over moderate distances. Similarly, at a 20 m distance, the average intensity decreases further to 1892, representing about 91% of the intensity observed at 10 m. Surprisingly, doubling the distance from 10 m to 20 m only results in a 9% decrease in intensity, indicating the resilience of LiDAR technology in preserving signal integrity over longer distances. These observations underscore an inverse relationship between distance and intensity values captured by 2LRLiDAR. Closer distances generally yield higher intensity values, while increasing distance reduces intensity due to signal attenuation. This analysis emphasizes the significance of considering distance when interpreting LiDAR intensity data for traffic sign detection and recognition applications.

## 5. Conclusions

This study thoroughly explores the impact of traffic sign visibility and LiDAR intensity on road safety in the context of AVs. The research uses advanced analysis methods facilitated by Python programming and statistical techniques to investigate the relationship between traffic sign retroreflectivity coefficients and LiDAR intensity. The study reveals differences and variabilities in retroreflectivity performance among RA1 and RA2 traffic signs, with implications for improving road safety standards and signage optimization. The key findings of the study are as follows:

- Variations in retroreflectivity performance between RA1 and RA2 traffic signs are significantly revealed, with RA2’s mean retroreflective coefficient of 263 notably higher than RA1’s mean coefficient of 37.
- The study highlights RA2’s greater resistance to weather conditions compared to RA1, with 87.8% of RA2 signs meeting standards compared to only 54.1% of RA1 signs.
- Analysis of color variations indicates that gray signs have the highest non-compliance rate of the specified standards outlined in the European standard EN 12899-1 at 71.4%, followed by white signs at 43.8%. Conversely, red and blue signs show the lowest non-compliance rates at 7.9% and 6.3%, respectively, followed by green and yellow signs.
- Statistical analysis reveals no significant difference in mean LiDAR intensity between RA1 and RA2 signs, suggesting comparable intensities within the observed data.
- RA2 signs generally exhibit higher mean intensities and retroreflectivity coefficients than RA1 signs, suggesting a potential correlation between higher-intensity readings and increased retroreflectivity.
- Analysis of LiDAR intensity data and retroreflectivity coefficients for signs categorized by the presence or absence of white coloration indicates slight variations between the two groups, suggesting that white-colored signs generally possess higher retroreflective properties.
- Variations in intensity readings between the two LiDAR systems could be attributed to differences in technology, calibration methods, hardware, processing algorithms, or environmental conditions. Notably, the correlation between intensity values of LiDAR systems and traffic sign retroreflectivity coefficients highlights differences in consistency between 2LRLiDAR and 1CLiDAR intensity measurements and their correlation with retroreflectivity coefficients.
- Average intensity decreases with increasing distance from the traffic sign. However, the decrease in LiDAR intensity is much less than the increase in distance.
- Mean intensity readings for traffic signs and their surrounding areas are contrasted, revealing a substantial difference in intensity levels. Traffic signs exhibit a mean intensity of 2192 with moderate variability, whereas surrounding areas have a mean intensity of 625 with a broader range of intensity values.
- The intensity difference between traffic signs and surroundings significantly affects LiDAR’s ability to detect and recognize traffic signs accurately, impacting safety and disrupting traffic management systems, particularly for AVs.
- LiDAR systems can well discriminate higher-intensity signals from traffic signs amidst lower-intensity backgrounds, enhancing detection accuracy and facilitating their applicability in detection processes.
- A limited correlation is observed between LiDAR intensity and handheld retroreflective coefficients, highlighting challenges posed by inherent differences in measurement methodologies and the need for further investigation and implementation of further quality control measures.

These findings collectively highlight the complexity of assessing traffic sign visibility and reflectivity while emphasizing the necessity for meticulous monitoring, quality control measures, and further research to address methodological challenges and enhance measurement accuracy.

Moving forward, the study proposes several recommendations:

- Implement quality control protocols to maintain consistent retroreflectivity performance across traffic sign categories.
- Explore additional factors influencing retroreflectivity and LiDAR intensity measurements to enhance accuracy and reliability.
- Investigate the practical implications of these findings on road infrastructure management and safety enhancement initiatives for informed decision-making.

Advancements in measurement techniques and standardization efforts hold promise for improving traffic sign visibility assessments and enhancing road safety standards. A nuanced understanding of factors influencing retroreflectivity and LiDAR intensity measurements may inform the development of more effective signage materials and maintenance strategies. Ultimately, the insights gleaned from this study could significantly contribute to optimizing road infrastructure management practices and facilitating the development of evidence-based road safety policies.

## 6. Limitations

The methodologies used in this study have some limitations, particularly concerning measurement techniques and their applicability for comparison.

Firstly, the retroreflectivity measurements used in this research simulate night driving conditions, where traffic signs reflect the light from vehicles’ headlights. However, it is essential to note that these coefficients are irrelevant when assessed under daylight conditions. In scenarios where signs are directly illuminated by sunlight or scattered light from the environment, the simulated night driving conditions do not accurately represent real-world visibility dynamics.

Secondly, it is essential to distinguish the different purposes served by retroreflectivity and LiDAR measurements. On the one hand, retroreflectivity measurements primarily serve the function of quality control and color recognition, and they take several minutes in a fixed position. LiDAR, on the other hand, helps detect signs but operates within a completely different paradigm. It is installed in moving vehicles and generates a point cloud within fractions of a second. Furthermore, there is a significant difference that exists in the spectral domains used by these methods: retroreflectivity measurements rely on normal light, specifically illuminant A, with a correlated color temperature of approximately 2856 Kelvin [25,26]. In contrast, LiDAR predominantly uses lasers within the near-infrared spectrum, typically ranging from 760 to 1900 nm in wavelength [20]. As a result, the limited correlation between these two measurement techniques is not surprising due to these fundamental differences in methodology and operating principles.

Additionally, AVs typically have camera systems, which are essential for recognizing the content of the signs. However, it is important to clarify that this research’s analysis did not include evaluating camera images, which represents a potential limitation in comprehensively assessing the efficacy of sign detection methodologies.

## Figures and Tables

**Table 1:** Retroreflectivity coefficient RA (cd.lx^−1^.m^−2^) for sheet class RA1 and RA2.

Sheet Class	Geometry of Measurement		Color								
	α	β1 (β2 = 0)	White	Yellow	Red	Green	Dark Green	Blue	Brown	Orange	Gray
**RA1**	20′	+5°	50.0	35.0	10.0	7.0	-	2.0	0.6	20.0	30.0
**RA2**	20′	+5°	180.0	120.0	25.0	21.0	14.0	14.0	8.0	65.0	90.0

α = observation angle and β = entrance angle.

**Table 2:** Evaluating retroreflective coefficients with standard specifications.

Compare to Standards	Traffic Sign Sheet Class			
	RA1		RA2	
	No.	%	No.	%
**Conform to standards**	60	54.1%	43	87.8%
**Out of standard ranges**	51	45.9%	6	12.2%
**Total**	111	100.0%	49	100.0%

**Table 3:** Analysis of non-conforming retroreflective coefficients of sign elements.

Traffic Sign Face	Traffic Sign Sheet Class			
	RA1		RA2	
	No.	%	No.	%
**Legend**	34	66.7%	6	100.0%
**Background**	15	29.4%	0	0.0%
**Background and legend**	2	3.9%	0	0.0%
**Total**	51	100.0%	6	100.0%

**Table 4:** Analysis of non-conforming retroreflective coefficients by colors.

Traffic Sign Color	Traffic Sign Sheet Class							
	RA1				RA2			
	Signs Conforming to Standards.	Signs out of Standard Ranges	Total	Rate (%)	Signs Conforming to Standards	Signs out of Standard Ranges	Total	Rate (%)
**White**	50	39	89	43.8%	42	6	48	12.5%
**Blue**	59	4	63	6.3%	32	0	32	0.0%
**Red**	58	5	63	7.9%	11	0	11	0.0%
**Gray**	2	5	7	71.4%	2	0	2	0.0%
**Green**	0	0	0	0.0%	4	0	4	0.0%
**Yellow**	0	0	0	0.0%	1	0	1	0.0%

**Table 5:** Analysis of retroreflective coefficients for traffic sign elements.

Traffic Sign Elements	No. of Signs	Retroreflectivity Coefficients		
		Mean	Standard Deviation	Relative Standard Deviation
Legend	160	122	153	125%
Background	160	90	152	168%
Average of legend and background	160	106	126	119%

**Table 6:** Independent samples *t*-test.

	*t*-Test for Equality of Means						
	t	df	Sig. (Two-Tailed)	Mean Difference	Std. Error Difference	95% Confidence Interval of the Difference	
						Lower	Upper
**Equal variances assumed**	1.87	318	0.06	31.87	17.03	−1.63	65.37

**Table 7:** Analysis of retroreflective coefficients for RA1 traffic sign elements.

RA1 Signs	No. of Signs	Retroreflectivity Coefficients		
		Mean	Standard Deviation	Relative Standard Deviation
**Legend**	111	41	33.2	80%
**Background**	111	33	32.1	97%
**Average of legend and background**	111	37	25.0	67%

**Table 8:** Analysis of retroreflective coefficients for RA2 traffic sign elements.

RA2 Signs	No. of Signs	Retroreflectivity Coefficients		
		Mean	Standard Deviation	Relative Standard Deviation
**Legend**	49	306	158.8	52%
**Background**	49	221	221.9	101%
**Average of legend and background**	49	263	123.6	47%

**Table 9:** Analysis of 2LRLiDAR intensity for traffic sign sheet classes.

Category	No. of Signs	2LRLiDAR Intensity		
		Mean	Standard Deviation	Relative Standard Deviation
**All signs**	60	2192	519.8	24%
**RA1**	53	2168	540.7	25%
**RA2**	7	2374	281.5	12%

**Table 10:** Analysis of 2LRLiDAR intensity for traffic sign by color composition.

Category	No. of Signs	2LRLiDAR Intensity		
		Mean	Standard Deviation	Relative Standard Deviation
**All signs**	60	2192	519.8	24%
**WB**	23	2260	579.6	26%
**WR**	18	2129	557.9	26%
**WG**	3	2233	150.1	6.7
**RB**	15	2154	461.6	21%
**WSN**	45	2205	542.0	25%

**Table 11:** Analysis of 1CLiDAR intensity for traffic sign sheet classes.

Category	No. of Signs	1CLiDAR Intensity		
		Mean	Standard Deviation	Relative Standard Deviation
**RA1**	69	1639	33.3	2.0%
**RA2**	36	1662	8.9	0.5%

**Table 12:** Analysis of 1CLiDAR intensity for traffic sign by color composition.

Category	No. of Signs	1CLiDAR Intensity		
		Mean	Standard Deviation	Relative Standard Deviation
**All signs**	105	1647	29.5	1.8%
**WB**	45	1649	28.7	1.7%
**WR**	33	1640	32.3	2.0%
**RB**	15	1657	16.7	1.0%
**WSN**	90	1645	31.0	1.9%

**Table 13:** LiDAR intensity comparison—traffic signs versus surrounding areas.

Category	No.	Surrounding Intensity		
		Mean	Standard Deviation	Relative Standard Deviation
**Traffic signs intensity**	60	2192	519.8	24%
**Surrounding area intensity**	60	625	391.0	63%

**Table 14:** Retroreflectivity coefficients and 2LRLiDAR intensity comparison by sheet classes.

Category	No. of Signs	2LRLiDAR Intensity			Retroreflectivity Coefficients		
		Mean	Standard Deviation	Relative Standard Deviation	Mean	Standard Deviation	Relative Standard Deviation
**All signs**	60	2192	519.8	24%	59	70	119%
**RA1**	53	2168	540.7	25%	40	29	73%
**RA2**	7	2340	281.5	12%	187	126	68%

**Table 15:** Retroreflectivity coefficients and 2LRLiDAR intensity comparison by colors.

Category	No. of Signs	2LRLiDAR Intensity			Retroreflectivity Coefficients		
		Mean	Standard Deviation	Relative Standard Deviation	Mean	Standard Deviation	Relative Standard Deviation
**All signs**	60	2192	519.8	24%	59	70	119%
**WSN**	45	2205	542.0	25%	71	77	109%
**SNW**	15	2154	461.6	21%	23	14	60%

**Table 16:** Retroreflectivity coefficients and 1CLiDAR intensity comparison by sheet classes.

Category	No. of Signs	1CLiDAR Intensity			Retroreflectivity Coefficients		
		Mean	Standard Deviation	Relative Standard Deviation	Mean	Standard Deviation	Relative Standard Deviation
**All signs**	105	1647	29.5	1.8%	127	142	111%
**RA1**	69	1639	33.3	2.0%	92	126	137%
**RA2**	36	1662	8.9	0.5%	296	118	40%

**Table 17:** Retroreflectivity coefficients and 1CLiDAR intensity comparison by color.

Category	No. of Signs	1CLiDAR Intensity			Retroreflectivity Coefficients		
		Mean	Standard Deviation	Relative Standard Deviation	Mean	Standard Deviation	Relative Standard Deviation
**All signs**	105	1647	29.5	1.8%	127	142	111%
**WSN**	90	1645	31.0	1.9%	144	146	102%
**SNW**	15	1657	16.7	1.0%	25	13	52%

**Table 18:** Quantification of 2LRLiDAR intensity at varied distances from traffic signs.

No. of Traffic Signs	Average LiDAR Intensity		
	Distance between 2LRLiDAR and Traffic Sign		
	10 m	15 m	20 m
**1**	1907	1886	1886
**2**	2010	1915	2023
**3**	2089	1716	1983
**4**	2307	2156	2022
**5**	2217	2028	1888
**6**	2232	2117	1952
**7**	2146	2010	1827
**8**	1999	1931	1931
**9**	2367	2303	2032
**10**	1545	1650	1389
**11**	2017	2028	1886
**Average**	2076	1976	1892
	100%	95%	91%

## Data Availability

Data are contained within the article.
